# Barriers and Facilitators to Sexual and Reproductive Healthcare for Women in Opioid Use Disorder Recovery: Perspectives from Recovery Healthcare Professionals

**DOI:** 10.3390/healthcare14162448

**Published:** 2026-08-07

**Authors:** Jessica L. Zemlak, Lisa Grabert, Hannah Brennan, Nicole Mattson

**Affiliations:** 1 College of Nursing, Marquette University, Straz Hall, 520T, P.O. Box 1881, Milwaukee, WI 53201, USA; 2 School of Nursing, Milwaukee Area Technical College, Milwaukee, WI 53233, USA

**Keywords:** sexual health, reproductive health, opioid-related disorders, women’s health

## Abstract

Objective: Women in recovery from opioid use disorder (OUD) experience sexual and reproductive health disparities which worsen health outcomes and may negatively impact recovery. While in recovery programs, women engage with different healthcare professionals, but how these professionals provide sexual and reproductive healthcare (SRH) is not well known. The purpose of this study is to identify perceived barriers and strategies to overcome barriers to providing SRH to women in recovery from OUD from the perspective of OUD recovery healthcare professionals. Methods: Guided by the social ecological framework, we used a qualitative descriptive study design to conduct individual semi-structured interviews with healthcare professionals (nurses, doctors, leaders/managers, peer support specialists, therapists, and case managers). Data was analyzed using thematic analysis. Results: Among individual interviews with *N* = 34 healthcare professionals, we identified two overarching themes: Barriers and Facilitators. Barriers included unmet provider training needs, navigating patient stigma and shame surrounding SRH, and fragmented healthcare systems. Facilitators included leveraging communities of shared experiences and developing ideal models of SRH programming. This included co-location of SRH and recovery spaces and the importance of peer support specialists in women’s recovery journeys. Conclusions: Multi-level challenges to address SRH in recovery require discipline specific training of healthcare professionals, empowering peer professionals, and policy and advocacy to address systemic issues.

## 1. Introduction

Women represent a growing number of the over 2.5 million people with an opioid use disorder (OUD) in the U.S [1] (). While there was a 265% increase in prescription opioid overdoses for men between 1999–2010, women had a 400% increase; indicating the growing need to better understand the needs of women with OUD [2,3]. Women also represent a growing population of individuals in recovery from OUD—engaging in treatment programs, support processes, and medication management to stop or limit opioid use [4]. Despite the increasing numbers of women in recovery, there is a lack of gender-specific treatment tailored for the unique needs of women in recovery (i.e., motherhood/care giving, experiences of intimate partner and sexual violence, and sexual/reproductive health) [5].

The lack of gender-specific sexual and reproductive health (SRH) support for women in recovery is particularly problematic due to health disparities such as higher rates of unintended pregnancy, sexual violence, and sexually transmitted infections [6]. The lack of tailored services for women is challenging because sexual health experiences such as sexual violence are associated not only with worse reproductive health outcomes (e.g., unintended pregnancy, sexually transmitted infections) but also lower rates of substance use treatment completion [7].

While in recovery, women often recognize and prioritize SRH health needs but also acknowledge challenges in meeting these needs. Previous research among women in recovery found that women endorse a holistic view of SRH inclusive of healthy relationships, prioritization of preventative healthcare, and accessible family planning services [8,9]. While women describe recovery as a time to refocus on SRH, barriers such as caregiving and societal issues such as stigma often impede women’s ability to meet their SRH needs in recovery [9,10]. Structural factors such as transportation and complexity of the healthcare system are also commonly described as barriers to obtaining SRH services while in recovery [4,6]. Women describe the value of gender-specific care and integration of SRH and recovery services to address some of these care barriers [10].

Recovery professionals play a critical role in supporting women’s health throughout the recovery process. Women in recovery from OUD often engage in treatment with substance use disorder healthcare professionals such as physicians, nurses, nurse practitioners, case managers, social workers, and peer support specialists. Despite engaging with recovery professionals, barriers to meeting women’s SRH needs in recovery persist. Since recovery professionals are a critical component of women’s health during their recovery journeys, perspectives of barriers and facilitators of meeting SRH from substance use disorder (SUD) healthcare professionals are vital to addressing health needs. Insights from recovery healthcare professionals regarding individual, interpersonal, community, and societal level factors can inform the development of interventions designed to improve SRH access, service delivery, and health outcomes for women.

**Figure 1:**
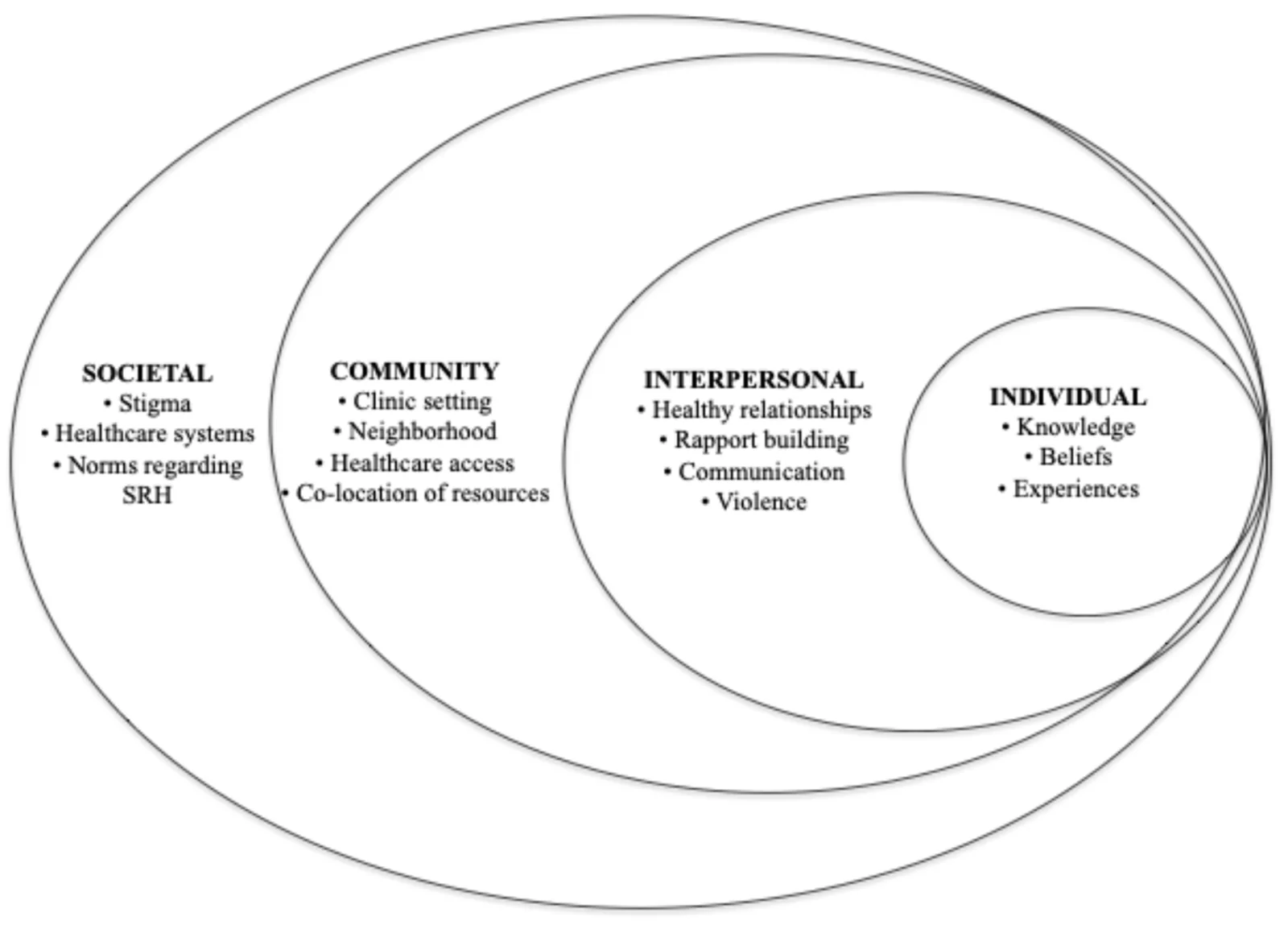
Sexual and Reproductive Health Concepts by Level of Social Ecological Framework.

Our research was guided by the social ecological framework allowing us to carefully consider these multiple levels of influence on providing SRH care to women in recovery [11]. The social ecological framework is commonly used in public health prevention research [12,13]. It is a multiple level framework that considers the interplay between individual, interpersonal, community, and societal factors impacting health [11]. We considered key focus areas at each level which might have implications for women’s SRH care in recovery (see Figure 1). We then used this framework in the development of our study interview guide and in shaping our understanding of our findings. Guided by the social ecological framework, the purpose of this study is to identify perceived barriers and strategies to overcome barriers to providing SRH to women in recovery from OUD from the perspective of OUD recovery healthcare professionals.

## 2. Methods

### 2.1. Design

We used a qualitative descriptive design for our study [14] and followed the Consolidated Criteria for Reporting Qualitative Study checklist in reporting our findings [15].

### 2.2. Participants

We purposively sampled healthcare professionals engaged in diverse roles who serve women in recovery from OUD in a variety of settings. Eligibility criteria included: (a) 18 or older, (b) currently work as a healthcare professional for women in recovery from OUD, (c) willing to participate in one telephone audio-recorded interview.

### 2.3. Recruitment

We recruited participants through local community partners and through snowball sampling approaches (participant word of mouth and sharing flyers with their professional networks). Study flyers were created with a brief description of the study, participant eligibility, a unique study telephone number and email address. Flyers were distributed through local community partners. Flyers were shared by these partners and participants with their professional networks and peers in national healthcare recovery groups. Through these approaches, our study recruited healthcare professionals from across the United States. Recruitment was conducted openly among healthcare professionals working in SUD recovery settings, and all interested individuals meeting eligibility criteria were invited to participate. As data collection progressed and preliminary analysis indicated that perspectives from certain professional roles were underrepresented, we engaged in targeted recruitment of individuals from those roles (e.g., peer support specialists and case managers) to increase the diversity of perspectives and voices represented in the sample and support thematic saturation across professional groups. Recruitment continued until sufficient depth and breadth of data was obtained and no new themes emerged. We assessed for meaning saturation through our weekly team meetings, analysis processes, and individual memo writing. The study was approved through the Marquette University institutional review board.

### 2.4. Instrument

We use the term “women/woman” to encompass all individuals who identify as women, regardless of sex assigned at birth. We acknowledge a critical gap in the literature, as research on SRH in recovery has largely centered on women assigned female at birth, with limited inclusion of transwomen’s experiences. Our interview guide in this study was created to gather provider experiences caring for all women regardless of their sex assigned at birth.

We developed our interview guide to align with the social ecological framework considering individual, interpersonal, community, and societal factors related to SRH and OUD recovery (see sample questions Table 1, and full interview guide in Supplementary Materials). When referencing SRH throughout the interview guide, we referenced and included language aligning with the World Health Organization’s holistic definition of sexual and reproductive health inclusive of sexual health (physical, emotional, and social well-being related to sexuality and sexual relationships) and reproductive health (family planning, reproductive justice, and safety in sex) [16]. We then sought feedback from healthcare professionals engaged with women in recovery to review our guide and it was revised prior to implementing with our participants.

### 2.5. Data Collection

Interested individuals emailed or called the research team and were screened for eligibility. If eligible, individuals were scheduled for an interview with a team member. Immediately prior to the start of the scheduled interview, the interviewer rescreened for eligibility, verbally read a consent form, and collected demographic data. All participants gave verbal consent. The interviewer entered demographic data into a Qualtrics survey in real time. We conducted one-time, individual audio-recorded telephone interviews with each participant using our theoretically grounded semi-structured interview guide. Interviews lasted between 45 minutes and 1.5 hours. Participants received a $75 gift card to an online vendor (Tango) to thank them for participating in the study. The Principal Investigators (PI)s (JZ and NM), nurses with extensive sexual and reproductive health recovery expertise, and trained in qualitative methods, conducted all interviews between March 2024 and May 2024.

### 2.6. Analysis

We used the Clark and Braun five-phase approach to thematic analysis with our data [17]. Three members of the research team analyzed the data. In the first phase, we familiarized ourselves with the data. We verified accuracy of our professionally transcribed transcriptions to our audio recordings. Our verified and de-identified transcripts were then uploaded to the Dedoose qualitative data analysis platform [18]. In the second phase, generating codes, we first created an a priori code book guided by the social-ecological model framework. The first three transcripts were coded by all team members using this codebook. We met weekly to discuss our experiences with coding and refine the code book and discuss coding disagreements until consensus on a coding decision was reached by at least 2 team members. Additional in vivo codes were developed, defined, and refined through an iterative process then added to the codebook. Once consensus was reached on the codebook, the remainder of the transcripts were then divided among coders. We kept memos to track our coding decisions, highlight areas where a second opinion was needed for coding decisions, and identify potential emerging patterns in the data. During the third phase, searching for themes, the members of the research team created a spreadsheet which details our initial patterns in the data and codes that fit with each pattern. Initial patterns were examined using a social ecological lens [11]. In the fourth phase, reviewing themes, our research team continued to refine initial themes to ensure coherent themes that answered the research question. In the fifth phase, defining and naming themes, we found that the identified barriers and facilitators spanned multiple levels of the theoretical framework rather than aligning with a single level. Therefore, themes were reported according to the overarching patterns identified in the data rather than by individual framework levels. In the final phase, producing the report, our team wrote a narrative summary of our findings.

### 2.7. Methodological Integrity

The first three transcripts were coded independently by each coder. Following independent coding, the three coders met to discuss agreement on coding. Our team used memo writing to keep track of decisions regarding developing themes and their relationships throughout the coding and analysis process.

### 2.8. Positionality

Our research team consisted of three cis-gender women of reproductive age. Our team consisted of two PhD-prepared registered nurses with trauma informed training and experience caring for women impacted by substance use and one graduate student with previous research experience in trauma informed research practices. We met regularly throughout all steps of the data collection and analysis process to discuss and engage in reflexive journaling to record how our personal history and biases may shape our analysis of the data.

## 3. Results

Among the *N* = 34 participants in the study (see Table 2), there were therapists (n = 13, 38%), leaders (individuals with supervisory roles such as medical director or clinic manager in addition to their healthcare professional role, n = 5, 15%), physicians (n = 5, 15%), registered nurses (n = 4, 12%), peer support specialists (n = 3, 9%), nurse practitioners (n = 2, 6%), and case managers/social workers (n = 2, 6%). We identified two overarching themes, Barriers and Facilitators. Subthemes emerged related to each overarching theme. Table 3 includes participants quotes related to Barriers and Table 4 includes participant quotes related to Facilitators.

### 3.1. Barriers

Health care professionals described individual, structural, and systemic societal barriers to the provision of SRH care for women in recovery from OUD. Across disciplines, there was a prioritization on holistic healthcare for women in recovery and acknowledgement of the growing complexity of caring for a diverse recovery population in an ever growing, complicated health care and social environment. Three subthemes emerged: recognized training needs, navigating stigma and feelings of shame among women in recovery, and the siloed nature of health care.

#### 3.1.1. Training Needs

While participants in our study represented a range of disciplines, there was a recognition across professional disciplines of a need for more training regarding SRH. The type of training participants desired aligned with their scope of practice. Participants recognized that people entering recovery have increasingly complex social situations related to gender identity, social and structural vulnerabilities, and caregiving responsibilities. The complexity of a diverse recovery population had implications in SRH care among OUD recovery professionals. Some healthcare professionals described receiving basic SRH training through their educational training, but it was often insufficient to meet the needs of the populations they serve in practice.

Participants described that due to the growing diversity in the population of women in recovery from OUD, there are now added skills related to SRH they needed in their practice. For example, healthcare professionals often desired more training regarding SRH when caring for transgender women. Many healthcare professionals filled some of these education or training gaps through on-the-job training/mentorship. Healthcare professionals often described peer professionals (e.g., peer support specialists, recovery coaches) as a potentially valuable resource for SRH needs of women in recovery. However, peer support specialists in our study felt they needed more training in SRH to meet these needs as this was not included in their formal training for their profession.

#### 3.1.2. Navigating Stigma and Feelings of Shame Among Women in Recovery

Many participants described that when discussing SRH with their patients, they heard about the pervasiveness of stigma and feelings of shame among their patients. When considering women and specifically SRH, professionals described uniquely complex issues surrounding stigma and women’s childbearing capacity. Most of the healthcare professionals in our study did not provide onsite comprehensive SRH services, thus there was a need to link women with other providers for some of their care needs. Participants described that when they discussed SRH care with patients or suggested a referral, they met resistance. Professionals often described the need to acknowledge and validate women’s descriptions of stigmatizing experiences. Thus, participants indicated they were often engaging in service recovery efforts to facilitate women accepting a referral or care coordination with necessary healthcare outside of the recovery space. Professionals often identified a need for SRH care for their patients, but also struggled with stagnant, stigmatizing beliefs among healthcare professionals outside of the recovery space.

Often, professionals are having these types of conversations when encouraging women to engage in necessary medical care such as SRH services. Professionals described that they often had conversations with patients about how the experience of caring for people in recovery who are mothers or have the capacity to become mothers adds a layer of complexity in supporting SRH. Some professionals described needing and wanting to challenge the stigma for women and mothers in recovery. Across disciplines, recovery healthcare professionals recognized experiences of stigma were barriers to addressing the SRH needs of women.

#### 3.1.3. Siloed Nature of Healthcare

A significant barrier described across provider disciplines was recognition that the U.S. healthcare system is systematically siloed with limited coordination between departments and specialties. The fragmentation separates SRH services and recovery care limiting access and long-term care coordination.

Most healthcare professionals worked in recovery spaces where co-location of recovery services and other health services on site did not exist. In these instances, patients needing SRH services would need referrals to other locations and professionals. Patients would be uncertain where to go for care, professionals were unclear where and how to link patients to care, and often care delays existed. Some healthcare professionals worked in recovery spaces with integration of select SRH services. However, even within these recovery care delivery models, there were barriers to SRH. Healthcare professionals often struggled with the complex healthcare system which is difficult for both professionals and patients to navigate. They described finding work-around processes to meet the needs of individual patients in the moment but acknowledged the need for systematic changes in healthcare integration to address these barriers.

**Table 3:** Barriers theme, subthemes, and exemplar quotes.

Theme	Subtheme	Socio-Ecological Framework Level	Supporting Quotes
Barriers	Training Needs	Individual level	“In my opinion [my training was], so low. In my family medicine residency training, maybe we had one lecture.” Participant 16, Physician “I think most of my training actually was outside of formal nursing training or NP training. Prior to nursing school, I was actually a volunteer at the organization that I work at now... That’s where I think most of my training came.” Participant 17, Nurse Practitioner “I don’t remember any formal training for that at all. I almost feel like it’s just something that’s it’s picked up on as I’ve been in the role and using things that I know to be true even from health class back in the day…” Participant 34, Therapist More training. The peer role is not standardized either statewide or certainly not nationally. You’ve got to provide the mandatory education and standards for peers to have.” Participant 25, Peer support specialist. “I found just more challenging because it was new to me, was having a person who did not identify as female who came in pregnant and being cognizant of what terms they preferred. how should we refer to bottom parts and what are your preferences to minimize gender dysphoria and enhance your comfort while also protecting you and baby?” Participant 16, Physician
	Stigma and feelings of shame	Interpersonal, Societal levels	“They [women in recovery] don’t get the same trust from their providers as a woman that doesn’t have a history of using drugs. They’re always under suspicion.” Participant 32, Therapist “I’m still seeing a lot of that stigma in society playing a role specifically against women in recovery... when they [health care providers] hear that they [women] have a substance use disorder, people shame them that it doesn’t make you a good mom, it doesn’t make you a good wife, and you have this disorder in your life, which contributes to those symptoms of substance use disorder. Participant 15, Therapist “I think part of it is understanding that women in recovery can be mothers. There’s just this assumption, this stigma out there that these women don’t have, I guess for lack of a better word, agency and intelligence regarding their own family planning.” Participant 28, Leader/Manager “I think it’s difficult in general to have the idea of a pregnant woman being on a medication like methadone, knowing that their children, their babies will need, require medical assistance as a result of the fact that the mom has been on methadone. I think there’s a lot of stigma around that in this area. Women run away from child welfare services and health care services because they are afraid of losing their kids.” Participant 10, Leader/Manager.
	Siloed Nature of Healthcare	Community and societal levels	“It goes back to how just separated medicine is. You sometimes see women just unsure of who to turn to or waiting months to see a primary care clinician, and so that’s why they don’t have access to their contraception, next thing you know they got pregnant accidentally, next thing you know, legally we have all the barriers around women’s right to choose and the complications that come with that.” Participant 16, Physician “I run into a lot of issues with clients not knowing even where to go, how to even look up for a provider, or even have resources and then maybe not knowing those resources even exist.” Participant 7, Therapist “I do both primary care and then my suboxone work, it’s one of the unfortunate limitations of my clinic right now is that historically, we have tried to separate primary care from our suboxone care.” Participant 22, Leader/manager “I think the biggest challenge is we do a lot of things onsite, but we don’t do everything onsite. If someone’s like, I want an IUD, we can’t give it to them in the office. We have to refer them somewhere else.” Participant 17, Nurse Practitioner

### 3.2. Facilitators

Facilitators were described as existing or envisioned factors to promote the provision of SRH services. Some healthcare professionals experienced a facilitator to providing SRH services in their current practice while other professionals described ways in which their current practice could be improved or changed to facilitate the provision of SRH services. We identified two themes as facilitators: valuing a community of shared experiences and envisioning ideal SRH programming.

#### 3.2.1. Valuing a Community of Shared Experiences

Healthcare professionals acknowledged that creating a sense of community for women to share SRH experiences or learn from peer professionals was important in overcoming stigma and engaging in care. There was acknowledgement that engaging in groups or discussions with peers who had similar experiences helped reduce feelings of shame.

Building safe communities in recovery was seen as helpful in supporting women’s engagement in SRH skill-building such as learning about healthy relationships. Often, these shared communities were most effective when they were women-only groups. Healthcare professionals acknowledged there was value in gender-specific women’s groups to create safe spaces for women to share SRH experiences, while recognizing the challenges of continuing to provide inclusive safe spaces.

#### 3.2.2. Envisioning Ideal SRH Programming

While some healthcare professionals described aspects of their practice that addressed SRH for women, others described ways they envision improvements in the current processes or systems to help women’s SRH needs. For some professionals, integration of care versus siloing of services were important to addressing the SRH of women. Others described that the provision of SRH and services in the same location helped not only address barriers to care access but also build confidence in women’s abilities to navigate systems which have often been stigmatizing in the past. Peer support specialists were identified as having potential of playing an important role in the SRH of women in recovery.

**Table 4:** Facilitators theme, subthemes, and exemplar quotes.

Theme	Subtheme	Socio-Ecologic Framework Level	Supporting Quotes
Facilitators	Community of shared experiences	Community level	“Where there are safe places for these women to go to, to be able to find these healthy relationships and be able to be emotionally vulnerable and find that support system that they need.” Participant 15, Therapist “The feeling of community as well, the feeling of belonging. When I work with women in recovery specifically, a lot of them, find their home group, like AA or NA, to be a women’s only group, which I feel like there is strength in that because there’s that sense of belonging there because of shared experiences.” Participant 15, Therapist “It’s helpful to have the women to have that space apart from men to feel safe. All of those groups, as long as those groups are including all women, including transgender women, I think those are generally really positive spaces.” Participant 27, Therapist “Professionally but also just socially and just in everything, [female-only groups] could be helpful. It comes up a lot more in female-only groups, women groups.” Participant 19, Therapist
	Envisioning ideal SRH programming	Individual, interpersonal, community, and societal levels	It would probably be just an integrated health system that had all the things. All the services were geared towards family, towards women, towards children, towards partner relationships, healthy sexuality, peer coaches, life coaches, therapists on an individual and group setting… Instead of trying to expand time and access, we find ourselves further siloing things in medicine, and I don’t think that that’s going to be the solution. Participant 16, Physician Exposing women to resources: I think it’s been really beneficial to have healthcare providers come on site... Okay, now I [the woman in recovery] know someone who’s a provider. Now I’ve had an experience that was positive. Now I’m more comfortable engaging in these services. Participant 4, Therapist. I think that peer support is really important because a peer support specialist has the knowledge and the tools to guide someone going through that. Participant 30, Therapist Peers, but, also individuals that have the extra training on how to access resources, things like that. Just that offering of that sense of community too, which I mean, it’s individualized, right? But I mean, that sounds like the perfect place to be for a woman in recovery is to be surrounded by peers, as well as those resources that they need. Participant 15, Therapist They come and I meet with them [women in recovery]... I remember walking in this place for treatment and it’s a really hard thing to do… I think it helps to have somebody that’s been there just let them know that this is a safe place, and show them around, and make contact with someone so that they have a familiar face so it’s more apt to bring them back. Participant 12, Peer Support Specialist

## 4. Discussion

Sexual and reproductive health (SRH) is a critical component of wellness for women in recovery from OUD. Our research, guided by the social ecological framework, deepened understanding of barriers and facilitators that the team of multidisciplinary OUD recovery healthcare professionals experience when considering SRH for their patients. We identified barriers and facilitators across individual, interpersonal, community, and societal levels which may have important implications research, practice, and policy change needed to improve SRH for women in recovery. Many professionals have incorporated processes to facilitate better provision of SRH care in their practice or have identified practical solutions to improve care for women. Our research, through the lens of direct patient care professionals, builds on previous studies among women in recovery identifying the care gaps and unique care needs for women in recovery.

### 4.1. Individual Level Implications

At the individual level, we identified training needs as a barrier to SRH care provision. Professionals across multiple disciplines commonly described the need for increased discipline relevant training on SRH. A few received limited formal training in their educational programming, and others received some on-the-job training. Many professionals described a lack of training regarding caring for transgender and gender diverse individuals in recovery. While physicians, nurses, and nurse practitioners in our study expressed a need for additional training in sexual and reproductive health (SRH), they also recognized that their clinical education and experience provide a strong foundation for expanding SRH competencies. Conversely, many therapists and case managers reported substantial training in healthy relationships and the psychosocial dimensions of SRH but felt less prepared to address reproductive health topics such as contraception and sexually transmitted infections. Peer support professionals often described confidence in their lived experience with SRH but acknowledged they had limited professional training on these topics. These findings suggest that each discipline brings complementary strengths that can be leveraged through interdisciplinary education and collaboration.

These gaps in training for recovery professionals are important to address as, for many women, their recovery professionals are their primary healthcare contacts. Women in recovery often do not seek other medical services outside of recovery due to factors such as stigma, transportation barriers, or complex caregiving needs may make accessing additional healthcare services challenging [19]. Previous research has found that women in recovery have identified gaps in their own knowledge about SRH [9]. Therefore, women may rely on their OUD professionals for SRH education and support. In previous research, women have endorsed a holistic definition of SRH to include healthy relationships, reproductive autonomy, fertility, and sexuality in recovery [9]. Training inclusive of this holistic definition of SRH is not formalized across healthcare professional disciplines, thus recovery professionals struggle to meet women’s health needs.

Training that acknowledges the unique discipline-specific and cross-disciplinary training on holistic SRH topics for OUD recovery professionals is essential in provision of holistic recovery care services [20]. While many professionals described receiving some form of basic SRH training in their education, most learned on the job. Their formal discipline educational training was broad and did not fully prepare them for the SRH needs of women in recovery. Thus, the development of SRH training programs for recovery professionals inclusive of holistic topics such as sexuality, healthy relationships, contraceptive options, gender-affirming care, and sexually transmitted infections are needed. Future research developing and implementing curricula to enhance confidence and capacity of recovery professionals in SRH topics may have downstream positive impacts on the health of women in recovery.

### 4.2. Interpersonal Level Implications

Many healthcare professionals described supporting women in recovery as they navigated feelings of shame related to sexual and reproductive health (SRH) and previous stigmatizing encounters with healthcare systems. Obstetrician-gynecologists are well positioned to provide trauma-informed, non-stigmatizing SRH care and may play an important role in integrated models of care [21]. By offering both medications for opioid uses disorder and gynecologic services, these providers have the potential to address recovery and SRH needs concurrently, reducing barriers to care and improving care coordination. However, only 2% of these providers are trained to provide medications for opioid use disorder [22]. Addressing SRH in recovery and improving patient/provider relationships requires that healthcare professionals, such as obstetrician gynecologists, receive training on the care of women in recovery.

Participants described a need to advocate for patients and provide supportive communication regarding SRH. Several participants identified peer professionals as having great potential of providing support to address these challenges. Peer Support Specialists (PSS), specially trained individuals (peers) with lived experience who provide support to individuals with mental health symptoms and/or recovery from substance use disorder, were described by many participants as important to improving SRH for women in recovery [23]. While PSS function in a variety of substance use treatment settings, their scope of practice and ethical guidelines clearly delineate the role as supportive and working with peers as equals [23,24]. The PSS has been shown to be effective in improving recovery experiences for pregnant people with SUD such as improved recovery [25]. The combination of the scope of practice of PSS along with lived experience may make supporting potentially sensitive areas of SRH during recovery (e.g., history of sex work) particularly valuable for women’s health and recovery journeys. While PSS undergo training and state-level certifications, that training is not uniform across the country. Therefore, additional research among PSS is needed to identify the critical knowledge, skills, abilities for PSS to be effective in supporting SRH for women in recovery.

### 4.3. Community Level Implications

A safe community for women to share experiences was an important facilitator of meeting SRH needs in recovery. Professionals described the sensitive nature of navigating SRH for women in recovery (e.g., discussing sexuality, intimate partner violence). Many professionals described gender-specific, woman only treatment spaces and groups as important to supporting women’s needs in recovery. Previous research among women in recovery also highlighted the value of gender-specific services in recovery [9,10]. However, as few as 20% of recovery centers have woman-specific programming [26]. Women represent a growing population of people in OUD recovery, therefore efforts within the recovery community must be made to create gender-specific, safe spaces and tailored services for women in recovery.

### 4.4. Societal Level Implications

A significant barrier to addressing SRH needs for women in recovery is systemic issues within the healthcare system itself. Policy changes are one tool that may break down some of the existing entrenched barriers siloing the healthcare system. Nurses are the largest discipline in healthcare and thus have great potential to be policy advocates for women in recovery. However, previous research has shown that nurses often become involved late in the process, during implementation, rather than during policy formulation [27]. There is a need to train, promote, and engage nurses in the policy process to be advocates for systemic healthcare changes.

One approach to engage more professionals earlier in the policy pipeline is during professional training and education. For example, American Academy of Colleges of Nursing which outlines competencies for professional nursing education has embedded policy across the curriculum [28]. This will bolster future nurses to engage earlier in the policy process and potentially have substantial impacts on the health issues such as those facing women in recovery. Similar integration of didactic policy training across health discipline education may help recovery professionals effect policy level changes with huge downstream health impacts.

Having recovery professionals educated and aware of policy may facilitate their ability to leverage policies to the benefit of their patients. Existing policies to address SRH needs within the OUD population have emerged at the federal level. On 24 October 2018, President Trump signed the *Substance Use Disorder Prevention that Promotes Opioid Recovery and Treatment for Patients and Communities* or the ‘‘SUPPORT” Act into law [29]. Section 7064 of SUPPORT provides resources to the Centers for Disease Control and Prevention (CDC) and states to collect, analyze, and make public data on substance abuse related to prenatal and postnatal health [29]. The recently enacted SUPPORT for Patients and Communities Reauthorization Act of 2025 or the “SUPPORT Extension” provides an additional $4.3 million (section 101) and four years of authorization (2026–2030) to continue making progress toward establishing the national database [30]. But lack of awareness and/or difficulty complying with the qualification criteria may probit some health professionals from accessing data and implementing funds.

Like the national database, section 7151, which focuses on building communities of recovery, was also recently extended (2026–2030) and allocated an additional $85 million (section 301) by the SUPPORT Extension Act [29,30]. Beyond access to data, the SUPPORT Act also includes funds that could aid SRH professionals. Specifically, section 7151 provides awards grants to nonprofit organizations who provide resources that target peer support networks to increase long-term recovery from OUD [29,30]. One potential barrier to accessing these dedicated funds is qualifying organizations were required to be “principally governed by people in recovery” [29,30]. This requirement meant SRH professionals needed to partner with community organizations to access resources. Section 7151 intended to address training needs of health professionals, address stigma, and expand services across several disciplines including behavioral health and primary care [29,30]. All of these areas were explicitly identified as barriers in our study.

## 5. Limitations

This study should be considered with some limitations. Our participants were from geographic regions around the United States, however, experiences and perspectives may vary based on state level differences in laws and reimbursement policies. Thus, participants from other states may have different perspectives. We used a convenience sampling approach which may not represent the broader views of recovery professionals. Our study only included perspectives of healthcare professionals and did not include perspectives of women in recovery as we were building on previous research exploring similar concepts among women. However, the decision to not include women in recovery in this study may limit our understanding of their experiences with barriers and facilitators of SRH care in recovery.

## 6. Conclusions

Sexual and reproductive health is a critical component of wellness for women in recovery from OUD. It is imperative to understand the unique perspective of barriers and facilitators experienced by various healthcare professional disciplines who work to optimize outcomes among women in recovery. All professionals interviewed recognized their discipline specific training needs, the necessity to overcome feelings of shame and experiences of stigma when caring for women in recovery, and the added challenges that the siloed nature of healthcare present as barriers to providing SRH care. Professionals described components of an ideal SRH program which included the need to build a supportive community of individuals with shared lived experience and the potential benefit of peer support for SRH. Continuing to create a less fragmented healthcare experience for women in recovery which includes a holistic definition of SRH is essential to improve the health and wellbeing of women throughout their recovery journey. Nurses and healthcare professionals must join forces to challenge current policies related to reimbursement practices.

## Figures and Tables

**Table 1:** Theoretically grounded semi-structured interview guide sample questions.

Socio-Ecologic Level	Sample Questions
Individual	How do you define sexual and reproductive health? What additional training or knowledge would help you support SRH for women in recovery?
Interpersonal	What relationships do you think are important to women’s sexual and reproductive health in recovery? While working with women in recovery, how would you describe a healthy relationship to a woman?
Community	What are some ways you have seen community impact health? What SRH services or support do you see women having challenges receiving?
Societal	How do you think society’s views on recovery have changed over time? How do you think policies and laws impact SRH?

**Table 2:** Participant Demographics and Characteristics.

Characteristic		n (%)
Sex		
	Female	28 (82.4%)
	Male	6 (17.6%)
Gender		
	Woman	27 (79.4%)
	Man	7 (20.6%)
Age		
	18–30	8 (23.5%)
	31–40	13 (38.2%)
	41–50	3 (8.8%)
	51–60	7 (20.6%)
	Over 61	3 (8.8%)
Race		
	White	24 (70.6%)
	Black/African American	3 (8.8%)
	Asian/Pacific Islander	3 (8.8%)
	Latino	1 (2.9%)
	More than one race	3 (8.8%)
Time in Role		
	1–3 years	18 (52.9%)
	4–8 years	9 (26.5%)
	9–13 years	5 (14.7%)
	14 or more years	2 (5.9%)
Time Since Training		
	Still in training	4 (11.8%)
	Less than 5 years	14 (41.2%)
	5–10 years	6 (17.6%)
	11–20 years	5 (14.7%)
	Greater than 20 years	5 (14.7%)
Percent of Women Currently Served in Role		
	0–25	1 (2.9%)
	26–50	21 (61.8%)
	51–75	5 (14.7%)
	76–100	7 (20.6%)
Practice Region		
	Northeast	10 (29.4%)
	Midwest	22 (64.7%)
	South	0 (0.0%)
	West	2 (5.9%)
SRH Programming Offered at Practice Sites		
	Pregnancy Testing	27 (79.4%)
	HIV Screening	25 (73.5%)
	Hepatitis C Screening	22 (64.7%)
	Contraception	18 (52.9%)
	Emergency Contraception	10 (29.4%)
	Healthy Relationship Training	24 (70.6%)
	Pregnancy Case Management	19 (55.9%)
	Pre-Exposure Prophylaxis	11 (32.4%)
	Parenting Training	2 (5.9%)

## Data Availability

The raw data supporting the conclusions of this article will be made available by the authors on request.

## Supplementary Materials

The following supporting information can be downloaded at: https://www.mdpi.com/article/10.3390/healthcare14162448/s1, Interview guide supplement is the full interview guide instrument used in this study.
