# Supplementary material for: Barriers and Facilitators to Sexual and Reproductive Healthcare for Women in Opioid Use Disorder Recovery: Perspectives from Recovery Healthcare Professionals
Source: Healthcare (Basel). 2026 Aug 7;14(16):2448. doi: 10.3390/healthcare14162448 (PMC13512076; doi:10.3390/healthcare14162448)
Supplement: Supplementary file 1 [file healthcare-14-02448-s001.zip › healthcare-4382993-supplementary.pdf]

Qualitative Interview Guide

Interviewer:
Thank you for your interest in being part of this project. Your interview will help us learn more about the experience and perspective of providers when caring for women in opioid recovery. The information you share will be used in the future to help women improve their reproductive health.

Understanding sexual and reproductive (SRH) needs at the individual level: Questions in this section will focus on understanding individual level SRH needs such as healthy relationships, sexuality, dating, individual's knowledge and experiences with contraception and HIV prevention measures.

Interviewer: A focus of this study is sexual and reproductive health. The next several questions will help us understand how you think and feel about health.

First, can you tell me a bit about yourself. What is your professional role? What is your training or educational background? What is your current practice or setting?

- How do you define SRH?
- How do you see your role in discussing SRH with women in recovery? How would you describe your comfort level in discussing SRH with women in recovery in your role?
    - Can you tell me about your training to discuss SRH needs with patients?
    - What additional training or knowledge do you feel would enhance your ability to provide SRH services to women in opioid recovery?
- Tell me about a time when you had a discussion about SRH with a woman in recovery.
    - What went well?
    - What do you think could have been improved?
    - What topics did you discuss related to SRH?
- What ways do you incorporate SRH into your practice with women in recovery?
    - o What facilitates incorporating SRH care into your practice?
    - o What strategies do you use to educate and engage women in opioid recovery about their SRH?
    - o How do you approach long-term care and follow-up in SRH for women who have recovered from opioid use?
- What challenges, if any, do you believe exist for incorporating SRH care into your practice?
    - o Have you encountered unique challenges or needs when providing SRH services to specific sub-populations of women in opioid recovery, such as adolescents, older women, currently or recently pregnant, women involved in sex work or trafficking, or those from diverse cultural backgrounds?

Understanding SRH at the interpersonal level: Questions in this section will examine interpersonal level factors such as rapport building, communication, intimate partner violence experiences and interpersonal dynamics.

Interviewer: Relationships with partners, friends, and family can shape how many people think about their health. Relationships can change for many women over time.

- What relationships do you think are important to women's sexual health and recovery?

- While working with women in recovery, how would you describe a healthy relationship to a woman?

- What types of conversations have you had about violence and trauma with women in recovery?
    - What type of advice or guidance do you share related to relationships?

- What training, if any, do you provide on healthy relationships in your role/practice?
    - How do you provide it?

How do you talk to women about dating and sex in recovery? What about online dating?

- What challenges, if any, do you believe exist for incorporating care related to relationships and interpersonal dynamics into your practice?
- What kind of discussions to you have about safety planning in relationships? In Dating? In online dating?

- If you could envision an ideal program focused on healthy relationships, what would it look like?

Understanding SRH at the community level: Questions in this section will examine community level factors such as access to health care and treatment setting.
Interviewer:

- How do you describe community for women in recovery?
    - Is community a physical space?
    - Is it the people? Or is it both?
    - Why do you feel this way?
    - What about the role of technology or online spaces?
- What are some ways you have seen community impact health? Either positive or negative impacts.
- How do you describe your role in women's recovery community?
- Should women have SRH groups or education that is just for women? Why or why not?
- What SRH services or support do you see women have challenges receiving?
- In what ways have you seen a person's neighborhood impact their sexual and reproductive health?

    - How have women's neighborhoods (the neighborhoods they come from or go to) impacted your experiences providing care?

Understanding health at the societal level: Questions in this section will examine factors such as stigma and its impact on sexual and reproductive health.

Interviewer:

- How do you think society's views on recovery have changed over time?
- What do you wish society knew about women's sexual health in recovery?
- How do you think policies and laws impact SRH in recovery?
    - What policy changes or additional resources do you believe would most significantly improve SRH care for women in opioid recovery?
- In your experience, how does stigma related to opioid use affect women's access to and engagement with SRH services?
- Based on your experiences and observations, if you had an unlimited budget and unlimited time, what would a comprehensive program for SRH for women in recovery look like?

Closing question: This question is to allow participants to share anything they feel is important to help aid in understanding sexual and reproductive health.

Interviewer: Is there anything you would like to share with me that we did not discuss today that you think is important for me to understand your experiences and perspectives regarding sexual and reproductive health needs of women in recovery?
